# Association between serum folate concentrations and blood lead levels in adolescents: A cross-sectional study

**DOI:** 10.3389/fped.2022.941651

**Published:** 2022-10-25

**Authors:** Huan He, Zhan Zhang, Min Li

**Affiliations:** Department of Ultrasound, Xi’an Children’s Hospital, Xi’an, China

**Keywords:** serum folate, blood lead, adolescents, NHANES, a cross sectional study

## Abstract

As a heavy metal, lead is a common toxic agent. Its accumulation in the body is harmful to physical health, particularly in children and adolescents. Studies have reported that folate may play a protective role in lead exposure. An association between serum folate concentrations (SFC) and blood lead levels (BLL) has been documented in adults, but studies in adolescents are limited. This study investigated the relationship between SFC and BLL in American adolescents. This cross-sectional study collected relevant data on both SFC and BLL of 5,195 adolescents in the NHANES database from 2007 to 2018. Multivariable linear regressions and smooth curve fittings were adopted to evaluate the correlation between BLL and SFC. After adjusting potential confounders, we found negative relationships between BLL and SFC [*β* = −0.0041 (−0.0063, −0.0019)], and the associations were significant in non-Hispanic Whites, Mexican Americans, and other races but not significant in non-Hispanic blacks (*P* = 0.139). Furthermore, the negative trends were significant in adolescents aged 16–19 years and females aged 12–15 years but insignificant in males aged 12–15 years (*P* = 0.172). Therefore, these findings provide a basis for future research on the mechanism of folate in regulating blood lead levels.

## Introduction

With the rapid development of industrial globalization and technology, heavy metal pollution caused by these processes has become a global public health concern. Heavy metals have bioaccumulation effects and persist in the biosphere (1). Therefore, almost all organisms will be threatened by their toxicity. Lead, as one of the common heavy metals, widely exists in our living environment and can enter the human body in many forms, such as drinking water, air, food, and daily necessities (2). Lead can also cause damage to many systems of the human body, among which hematopoietic, nervous, circulatory, and urinary systems are more sensitive to lead toxicity (3–5). Children and adolescents are at the stage of growth and development, with strong absorption capacity and low elimination rates of lead, and are more vulnerable to lead toxicity during these ages (6). In the past 50 years, the United States has significantly reduced childhood exposure to lead by not using leaded gasoline and paint (7). However, many children are still at risk of exposure and lead poisoning. At present, no blood lead level is considered safe. According to the latest data from the 2011–2014 national health and Nutrition Survey (NHANES), the CDC has the reference value of children's blood lead from 5 μg/dl decreased to 3.5 μg/dl (8). Unfortunately, low blood lead concentrations (mean 1.7 ± 0.3 μg/dl) can still harm children's intellectual development and overall blood function (9).

Folic acid is an essential water-soluble vitamin for the human body. It can not be synthesized by the human body and is obtained through food and vitamin/mineral consumption. Common foods rich in folic acid include leafy vegetables, beans, nuts, fruits, and berries (10). Studies have shown that a certain amount of folic acid intake by adolescents has a positive effect on improving cognition, promoting the development of the nervous system, and reducing anemia (11–13). Epidemiological studies show that the serum unsaturated fatty acids and folic acids of adolescents with depression are lower than those of adolescents with normal development (14). In another study, after long-term oral administration of 3.5 mg folic acid and 60 or 120 mg iron every week, the average hemoglobin in adolescent girls continued to increase with added treatment time (15).

The harm of heavy metals to the human body is primarily caused by oxidative stress (16). Previous studies have found that glutathione (GSH) can alleviate the oxidative stress induced by heavy metals and combine with heavy metals to form a complex, increase the excretion of heavy metals, and alleviate the harm of heavy metals to the human body (17). GSH is the precursor of cysteine. Interestingly, folic acid can directly pass through the trans-sulfur pathway and participate in the anabolism of cysteine and that in turn might increase GSH levels (18). Therefore, folic acid may participate in the detoxification process of heavy metals. A previous cross-sectional study has found negative relationship between lead exposure and serum folate in American adults (19). However, the research on the relationship between serum folate concentrations (SFC) and blood lead levels (BLL) in adolescents is limited. Therefore, exploring the relationship between BLL and SFC in adolescents is necessary, which may be significant in preventing lead poisoning in adolescents. This study used a nationally representative cross-sectional sample to explore the relationship between SFC and BLL in American adolescents. Furthermore, we further explored the relationship between SFC and BLL in the grouping of race, sex, and age to evaluate potential interactions between these factors.

## Materials and methods

### Study design and participants

In this study, data were collected from the National Health and Nutrition Examination Survey (NHANES) public database, which was part of the Centers for Disease Control and Prevention (CDC) in the United States. This database used a complex, stratified, multistage sample design to provide nationally representative data. Sources of data included interviews, medical examinations, and laboratory tests. Further methodological details about the NHANES survey were available at www.cdc.gov/nchs/nhanes/.

**Figure 1:**
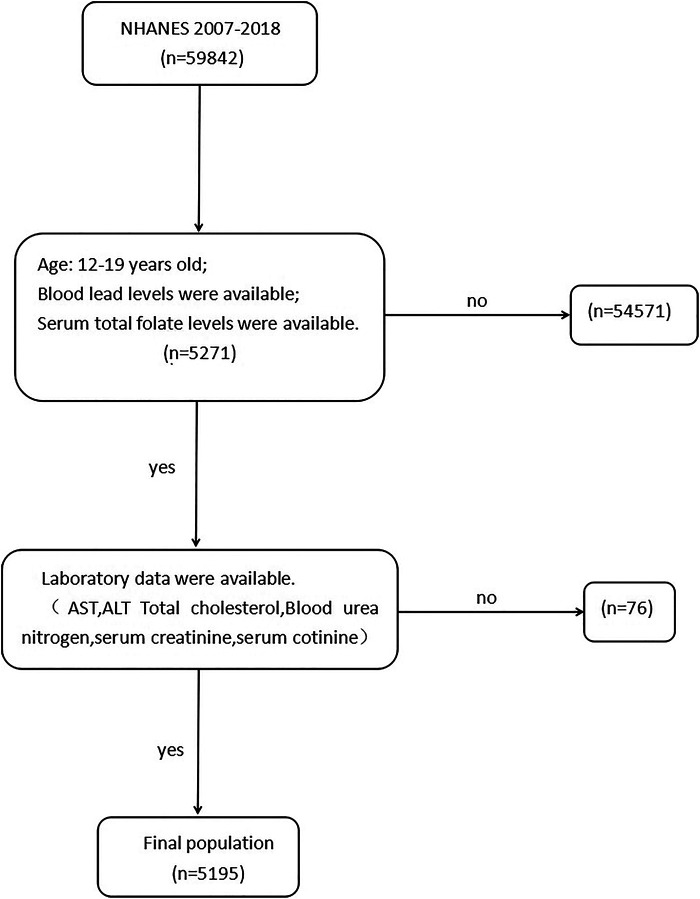
Flow chart of participants from NHANES included in the study. AST, aspartate aminotransferase, ALT, alanine aminotransferase.

A total of 59,842 subjects from six continuous cycles of the NHANES database were collected from 2007 to 2018. According to the inclusion criteria (Figure 1), 5,271 participants aged 12–19 years with complete data on SFC and BLL were included. Of the remaining 5,271 participants, we further excluded the population with missing laboratory data (*n* = 76). Finally, data from 5,195 participants were included in the study.

The NCHS Research Ethics Review Board approved all the procedures (20), and all the participants provided written informed consent. For participants under 18 years old, their parents/guardians provided informed consent.

### Study variables

#### Blood concentrations of serum folate and lead

Whole blood specimens were processed, stored, and shipped to the Division of Laboratory Sciences, National Center for Environmental Health, and Centers for Disease Control and Prevention for analysis.

SFC were measured using a microbiological assay (MBA) in NHANES from 2007 to 2010 (21).

From 2011 to 2018, SFC were measured by isotope-dilution high-performance liquid chromatography coupled to tandem mass spectrometry (LC-MS/MS). For total serum folate, the weighted distributions of the NHANES 2011–2012 data measured by LC-MS/MS were similar to the NHANES 2007–2008 and 2009–2010 data measured by MBA (22).

The method directly measured the lead content of whole blood specimens using inductively coupled plasma dynamic reaction cell mass spectrometry (ICP-DRC-MS) after a simple dilution sample preparation step (23). The lower detection limit for blood lead in NHANES from 2013 to 2018 was 0.07 µg/dl, and the lower detection limit for blood lead in NHANES from 2011 to 2012 was 0.25 µg/dl. (In cases where the result was below the detection limit, the value for that variable was the detection limit divided by the square root of 2.)

#### Other variables

The covariates were selected based on established associations (19, 24) and/or plausible biological relations to the variables examined. The following covariates were included in our study: age, sex, race, BMI, PIR (Ratio of family income to poverty), AST (aspartate aminotransferase), ALT (alanine aminotransferase), total cholesterol, blood urea nitrogen, serum creatinine, serum cotinine, and folate supplements. Details of these covariate acquisition processes were available at www.cdc.gov/nchs/nhanes/.

The body mass index (BMI) was categorized into three groups according to WHO standards: (i) <25 kg/m^2^, (ii) 25–30 kg/m^2^, and (iii) ≥30 kg/m^2^. The age of the participants was categorized into two groups: (i) 12–15 years and (ii) 16–19 years. Race was classified as “non-Hispanic Black”, “non-Hispanic White”, “Mexican American”, or “Other race”. PIR(Ratio of family income to poverty), AST, ALT, total cholesterol, blood urea nitrogen, serum creatinine, serum cotinine, and folate supplements were designed as continuous variables. In stratified analyses, these continuous variables were divided into three subgroups on average.

### Statistical analysis

In this study, all continuous variables were expressed as mean ± standard deviation, and the difference test between groups was calculated by a weighted linear regression model. Meanwhile, categorical variables were expressed as percentages, and the difference test of groups was calculated by weighted chi-square. Logistic regression models were applied to estimate the independent correlation between BLL and SFC before or after the adjustment of confounders. In each model, a weight suggested by the CDC was used to consider the oversampling of minorities and thus provide an accurate estimate of effects on the population. Weighted multivariate logistic regression models were used to explore the relationships between BLL and SFC. For this study, three sequential models (model 1: non-adjusted model; model 2: adjusted for age, sex, and race; model 3: adjusted for all covariates) were used to control potential confounders. A generalized additive model and smooth curve fitting were used to show the linear and non-linear relationship between the BLL and SFC. A *P*-value (*P*) ≤ 0.05 indicated a difference achieving statistical significance. The investigations were carried out using Empower software (www.empowerstats.com; X/Y Solutions, Inc., Boston, MA) and R version 3.4.3 (http://www.R-project.org, The R Foundation).

## Results

Five thousand one hundred ninety-five adolescents aged 12–19 were included in this study, including 48.63% males and 51.37% females. Among them, 56.30% were non-Hispanic Whites, 13.47% were non-Hispanic Blacks, 14.33% were Mexican Americans, and 15.90% were other races. The average BLL and SFC were 0.70 μg/dl and 18.88 ng/ml, respectively. Adolescents with lower serum total folate concentrations were more likely to be male, non-Hispanic Black, had higher serum cotinine levels, and had a lower PIR. As expected, participants who took folate supplements had higher folate levels. The lead levels in the blood decreased with the increase in serum folate concentrations (Table 1). Furthermore, blood lead concentrations were higher in non-Hispanic Black populations, those with higher serum cotinine levels, higher serum creatinine, and those from lower PIR (Supplementary Table S1).

**Table 1:** Characteristics of the study population based on serum folate concentration tertiles.

Serum folate concentration (ng/ml)	Total	T1 (2.72–13.70)	T2 (13.80–20.60)	T3 (20.70–79.00)	*P* for value
Age (years)	15.49 ± 2.24	16.22 ± 2.06	15.55 ± 2.21	14.83 ± 2.21	<0.0001
Sex (%)					0.0690
Men	48.63	50.28	49.38	46.56	
Women	51.37	49.72	50.62	53.44	
Race (%)					<0.0001
White	56.30	50.79	55.48	61.69	
Black	13.47	19.12	14.23	8.02	
Mexican American	14.33	13.98	14.82	14.18	
Other race	15.90	16.11	15.46	16.12	
PIR	2.52 ± 1.65	2.37 ± 1.63	2.48 ± 1.61	2.69 ± 1.68	<0.0001
BMI (kg/m^2^)	23.97 ± 6.02	25.32 ± 6.52	24.22 ± 6.06	22.60 ± 5.20	<0.0001
AST (U/L)	23.46 ± 10.55	22.60 ± 11.60	23.56 ± 11.31	24.10 ± 8.66	0.0001
ALT (U/L)	19.04 ± 14.22	18.99 ± 12.10	18.95 ± 13.63	19.17 ± 16.26	0.8895
Total cholesterol (mg/dl)	157.24 ± 29.43	156.85 ± 29.17	156.98 ± 28.90	157.80 ± 30.10	0.5792
Blood urea nitrogen (mg/dl)	10.83 ± 3.42	10.99 ± 3.69	10.77 ± 3.31	10.75 ± 3.29	0.0835
Serum creatinine (mg/dl)	0.73 ± 0.19	0.78 ± 0.25	0.72 ± 0.16	0.68 ± 0.15	<0.0001
Serum cotinine (mg/dl)	13.96 ± 57.16	24.05 ± 78.35	12.53 ± 50.50	6.76 ± 36.92	<0.0001
Folate supplements (mcg)	389.77 ± 284.22	323.82 ± 236.15	338.12 ± 279.52	432.28 ± 291.47	<0.0001
Blood lead (ug/dl)	0.70 ± 0.63	0.74 ± 0.62	0.71 ± 0.58	0.66 ± 0.68	0.0003

Mean ± SD for continuous variables: the *P* value was calculated by the weighted linear regression model. (%) for categorical variables: the *P* value was calculated by the weighted chi-square test. Abbreviation: PIR, ratio of family income to poverty; BMI, body mass index; AST, aspartate Aminotransferase; ALT, alanine aminotransferase.

As shown in Table 2, in multivariate-adjusted linear regression analysis, the SFC was negatively associated with BLL across the three models (Model 1: *β* = −0.0040, 95% CI: −0.0061, −0.0019; Model 2: *β* = −0.0039, 95% CI: −0.0060, −0.0017; and Model 3: *β* = −0.0041, 95% CI: −0.0063, −0.0019). When folate concentration was classified as a categorical variable, the highest tertile was significantly negatively associated with the BLL in three models.

**Table 2:** Regression coefficient (95% confidence interval) for BLL by serum SFC in adolescents aged 12–19 years in the 2007–2018 NHANES survey (*n* = 5,195).

Tertiles of SFC	Model 1 *β* (95% Confidence Interval)	Model 2 *β* (95% Confidence Interval)	Model 3 *β* (95% Confidence Interval)
Total	−0.0040 (−0.0061, −0.0019)	−0.0039 (−0.0060, −0.0017)	−0.0041 (−0.0063, −0.0019)
Q1	Reference	Reference	Reference
Q2	−0.0348 (−0.0778, 0.0083)	−0.0329 (−0.0753, 0.0095)	−0.0274 (−0.0696, 0.0147)
Q3	−0.0855 (−0.1277, −0.0434)	−0.0771 (−0.1201, −0.0342)	−0.0775 (−0.1215, −0.0335)

Model 1, no covariates were adjusted. Model 2, age, sex, race were adjusted. Model 3, age, sex, race, ratio of family income to poverty, body mass index, aspartate aminotransferase, alanine aminotransferase, total cholesterol, blood urea nitrogen, serum creatinine, serum cotinine, folate supplements were adjusted.

**Figure 2:**
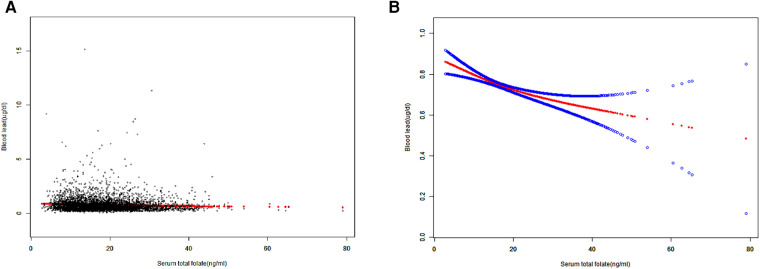
Smooth curve fitting of serum folate concentrations and blood lead levels. (**A**) Each black point represents a sample. (**B**) The area between two blue dotted lined is expressed as a 95% CI. The solid red line represents the smooth curve fit between the variables.Age, sex, race, ratio of family income to poverty, body mass index, aspartate aminotransferase, alanine aminotransferase, total cholesterol, blood urea nitrogen, serum creatinine, serum cotinine, folate supplements were adjusted.

When stratifying by age, sex or race, the negative correlation between SFC and BLL was significant in each subgroup of age and sex. However, in race subgroups, the relationship between SFC and BLL was significant in non-Hispanic Whites [−0.0037 (−0.0073, −0.0002)], Mexican Americans [−0.0065 (−0.0116, −0.0014)] and other races [−0.0076 (−0.0137, −0.0015)], but not significant in non-Hispanic black [−0.0034 (−0.0078, 0.0011)] (Table 3). Additionally, the negative trends were significant (*P*-trends <0.05) in adolescents aged 16–19 and female adolescents aged 12–15, while no associations were found between SFC and BLL in male adolescents aged 12–15 (Table 4). Furthermore, weighted generalized additive models and smooth curve fittings were used to visually assess the relationships between BLL and SFC, which were presented in Figure 2. The results showed that BLL was negatively associated with the SFC in the adjusted models.

**Table 3:** Association between SFC and BLL for stratified analyses according to baseline characteristics.

Subgroup	*N*	β (95% Confidence Interval)^a^	*P* for value
Age			
12–15 years	2,591	−0.0046 (−0.0074, −0.0018)	0.0011
16–19 years	2,604	−0.0042 (−0.0078, −0.0006)	0.0212
Sex			
Male	2,564	−0.0050 (−0.0085, −0.0014)	0.0057
Female	2,631	−0.0044 (−0.0072, −0.0016)	0.0023
Race			
Non-Hispanic white	1,499	−0.0037 (−0.0073, −0.0002)	0.0405
Non-Hispanic black	1,235	−0.0034 (−0.0078, 0.0011)	0.1392
Mexican American	1,192	−0.0065 (−0.0116, −0.0014)	0.0126
Other race	1,269	−0.0076 (−0.0137, −0.0015)	0.0146
PIR			
<1.05	1,575	−0.0070 (−0.0118, −0.0021)	0.0046
1.05–2.46	1,583	−0.0049 (−0.0084, −0.0014)	0.0060
≥2.46	1,585	−0.0034 (−0.0066, −0.0002)	0.0384
BMI (kg/m^2^)			
<25	3,297	−0.0054 (−0.0084, −0.0023)	0.0005
25–30	1,001	−0.0038 (−0.0074, −0.0003)	0.0357
≥30	824	0.0004 (−0.0048, 0.0055)	0.8946
AST (U/L)			
<20.00	1,636	−0.0048 (−0.0078, −0.0018)	0.0017
20.00–24.00	1,647	−0.0058 (−0.0093, −0.0023)	0.0013
≥24.00	1,912	−0.0042 (−0.0088, 0.0005)	0.0783
ALT (U/L)			
<14.00	1,481	−0.0058 (−0.0087, −0.0029)	<0.0001
14.00–19.00	1,935	−0.0042 (−0.0085, 0.0001)	0.0566
≥19.00	1,779	−0.0059 (−0.0097, −0.0022)	0.0020
Total cholesterol (mg/dl)			
<143.00	1,696	−0.0023 (−0.0068, 0.0022)	0.3184
143.00–167.00	1,762	−0.0042 (−0.0081, −0.0003)	0.0360
≥167.00	1,737	−0.0058 (−0.0089, −0.0027)	0.0002
Blood urea nitrogen (mg/dl)			
<9.00	1,407	−0.0025 (−0.0068, 0.0018)	0.2618
9.00–12.00	1,927	−0.0049 (−0.0079, −0.0020)	0.0012
≥12.00	1,861	−0.0040 (−0.0083, 0.0004)	0.0743
Serum creatinine (mg/dl)			
<0.62	1,509	−0.0027 (−0.0076, 0.0022)	0.2821
0.62–0.78	1,937	−0.0047 (−0.0077, −0.0017)	0.0021
≥0.78	1,749	−0.0061 (−0.0100, −0.0021)	0.0025
Serum cotinine (mg/dl)			
≤0.0190	1,675	−0.0018 (−0.0052, 0.0015)	0.2863
0.0190–0.1590	1,788	−0.0064 (−0.0097, −0.0031)	0.0001
≥0.1590	1,732	−0.0033 (−0.0081, 0.0014)	0.1666
Folate supplements (mcg)			
≤221.00	246	−0.0117 (−0.0263, 0.0030)	0.1211
221.00–476.00	253	−0.0050 (−0.0114, 0.0014)	0.1293
≥476.00	254	0.0072 (−0.0065, 0.0210)	0.3018

Abbreviation: PIR, ratio of family income to poverty; BMI, body mass index; AST, aspartate aminotransferase; ALT, alanine aminotransferase.

^a^ Models were adjusted for all covariates other than the stratification variable.

**Table 4:** Blood lead levels by tertiles of serum folate concentration, stratifified by age and sex.

Tertiles of SFC	BLL ug/dl (95% Confidence Interval)	
	Male	Female
**12–15 years**		
Lowest tertiles	1.0038 (0.9138, 1.0938)	0.6357 (0.5857, 0.6857)
2nd	0.8855 (0.8158, 0.9553)	0.6257 (0.5833, 0.6682)
3rd	0.9015 (0.8410, 0.9620)	0.5448 (0.5056, 0.5840)
*P* for trend	0.172	0.002
**16–19 years**		
Lowest tertiles	0.9135 (0.8605, 0.9665)	0.6456 (0.5878, 0.7033)
2nd	0.8662 (0.8059, 0.9264)	0.5822 (0.5217, 0.6427)
3rd	0.7117 (0.6290, 0.7944)	0.5428 (0.4729, 0.6126)
*P* for trend	<0.001	0.031

Age, race, ratio of family income to poverty, body mass index, aspartate aminotransferase, alanine aminotransferase, total cholesterol, blood urea nitrogen, serum creatinine, serum cotinine, folate supplements were adjusted.

## Discussion

The main objective of this study was to investigate whether SFC is independently associated with BLL in adolescents. This study suggests a negative association between serum folate concentrations and blood lead levels among adolescent participants aged 12–19 years from NHANES 2007–2018 survey. The association became more robust when the highest serum folate tertile group was compared with the lowest tertile group. To the best of our knowledge, our research is the first report investigating the relationship between lead levels and serum folate status in adolescents.

Previous studies have explored the association between lead and folate. A prospective birth cohort study suggests that adequate maternal folate status may mitigate the obesity risk associated with maternal lead exposure (25). Additionally, *in vitro* studies found folate deficiency to enhance DNA damage caused by exposure to heavy metals such as lead and chromium (26). Although the mechanism between lead and folate status was unclear, these findings may be attributed to the oxidative stress of lead and antioxidant properties of folate. Lead can easily cross the cell membrane, induce oxidative stress by disrupting the antioxidant system, and deplete intracellular glutathione (an important antioxidant) (27, 28). In contrast, folate can participate in glutathione anabolism through the transsulfuration pathway (29), which may reduce the toxicity caused by lead. Moreover, folate has also been reported to improve lead excretion and make it more difficult for lead to bind to blood elements, thereby reducing lead toxicity (30). Furthermore, animal experiments suggest that folate may antagonize the testicular toxicity of lead by increasing relative testicular weight and testosterone secretion, reducing oxidative stress and inflammatory responses, and down-regulating the NF-KB signaling pathway (31). Therefore, the above studies show that folate may participate in the metabolism of lead in the blood or reduce the harmful consequences of lead exposure. However, the underlying mechanism in detail requires further investigation.

In our study, the average BLL of the adolescents was 0.70 ug/dl, which was far below the safe levels used by the WHO and CDC (32). However, studies have shown that even relatively low levels of lead exposure are still associated with public health problems. Notably, a large body of literature has examined the effects of low levels of exposure of lead on the health of children and young adolescents. R.G. Lucchini et al. first demonstrated significant adverse cognitive effects of lead at low BPb levels (average BPb was 1.71 mg/dl) in adolescents aged 11–14 years (33). Low blood lead concentrations (1.8 ug/dl) are also associated with more autistic behaviors in school-age children (34). Meanwhile, Cho et al. found that children with a mean blood lead level of 1.9 μg/dl had symptoms of attention deficit hyperactivity disorder (ADHD) (35). An N-shaped curve was found between BLL and total BMD(bone mineral density), subtotal BMD and limb BMD for males and females at low-dose lead exposure (0.84 ug/dl) (36). In addition, when studying the relationship between blood lead and cardiovascular risk factors, Xu (24) found a positive correlation between BLL (geometric mean of 1.17 ug/dl) and serum LDL-C (low-density lipoprotein cholesterol, a biomarker associated with cardiovascular disease) in American adolescents. Therefore, the health effects of low levels of lead exposure cannot be ignored. Notably, further reducing BLL and preventing lead exposure is of great significance to the health of adolescents.

Currently, chelation therapy is the primary treatment that can aid in acute Pb poisoning (37). However, chelating agents can increase essential metals' excretion during treatment and have other potential side effects for children and adolescents (38). Therefore, it is essential to identify non-invasive natural treatments or compounds that might reduce BLL in children and adolescents. Our study showed a negative correlation between SFC and BLL, which indicates that folate may be involved in the detoxification of lead and may prevent low-dose chronic lead poisoning. Further prospective cohort studies are needed to confirm these findings.

Our study found that non-Hispanic blacks and subjects with low PIR have higher BLL and are more vulnerable to lead exposure. The possible explanation for these findings is due to social inequities that place ethnic minority adolescents at a higher risk for lead exposure (39, 40). On the other hand, poverty is another risk factor for lead exposure. Adolescents living in poverty are more likely to be exposed to lead-contaminated soil and to live near industrial facilities that emit lead pollutants (38). We also found that non-Hispanic blacks and subjects with low PIR have lower SFC, which is consistent with the conclusion in another NHANES cross-sectional study (41) that found Non-Hispanic blacks and subjects with lower socioeconomic status were accounted for the poor nutritional status of most biomarkers, including folate.

In subgroup analysis, although the negative association between BLL and SFC was insignificant in non-Hispanic blacks, we still found that non-Hispanic blacks with lower serum folate concentrations were likelier to have higher BLL. Previous cross-sectional studies found a negative association between BLL and SFC in different populations (19, 30). However, few studies have analyzed whether this relationship exists in different ethnic groups. This finding in the study suggests that the relationship may differ between races. We speculate that the variability may be due to other potentially confounding factors which are difficult to assess (e.g., exercise and dietary patterns). Among adolescents aged 12–15, we found that the negative trends were significant between BLL and SFC in females but not males. The sources of the sex differences between BLL and SFC are not known. It could be that hormonal differences between males and females mediated these findings. Alternatively, this could be a chance effect. Future studies should attempt to replicate these findings and if significant further explore these possibilities.

This is the first study to analyze adolescents' relationship between SFC and BLL. In this study, we selected a representative American adolescent population and conducted further stratified analyses, which is the advantage of our research. However, this study also has some noted limitations. Firstly, the current study is a cross-sectional study, and it cannot explain causal relationships. Further prospective research is needed in the future. Secondly, as in all observational studies, residual confounding is still possible despite our adjustment and stratification for various potential confounders. Thirdly, our study did not include the indicators used to measure the protective role of folate in lead exposure. For example, the increase in blood lead concentration can lead to anemia (42), and the hemoglobin content can reflect whether there is anemia. Therefore, it is unknown whether serum folate exerts a protective effect on the anemia caused by lead. Further research is needed in order to better understand whether and how serum folate decreases BLLs in adolescents and its impact on other important health conditions that have been associated with chronic lead exposure such as anemia.

## Conclusions

In conclusion, we found a negative correlation between SFC and BLL in American adolescents.

This association was insignificant in non-Hispanic blacks and males aged 12–15 years. These findings may provide new insights into the treatment of lead poisoning and the study of reducing blood lead levels in adolescents. Furthermore, potential differences in sex and races need more studies in the future to better explain these relationships and apply preventative measures.

## Data Availability

The datasets presented in this study can be found in online repositories. The names of the repository/repositories and accession number(s) can be found below: www.cdc.gov/nchs/nhanes/.
