# Supplementary material for: Association between serum folate concentrations and blood lead levels in adolescents: A cross-sectional study
Source: Front Pediatr. 2022 Oct 25;10:941651. doi: 10.3389/fped.2022.941651 (PMC9641282; doi:10.3389/fped.2022.941651)
Supplement: Supplementary file 1 [file Table1.docx]

Supplement Table 1: Characteristics of the study population based on blood lead levels tertiles

| blood lead levels(ug/dl) | Total | T1(0.33 ± 0.08) | T2(0.58 ± 0.08) | T3(1.27 ± 0.90) | P for value |
| --- | --- | --- | --- | --- | --- |
| Age (years) | 15.49 ± 2.24 | 15.48 ± 2.14 | 15.61 ± 2.23 | 15.38 ± 2.36 | 0.0123 |
| Sex (%) |  |  |  |  | <0.0001 |
| Men | 48.63 | 31.36 | 49.70 | 67.80 |  |
| Women | 51.37 | 68.64 | 50.30 | 32.20 |  |
| Race/ethnicity (%) |  |  |  |  | <0.0001 |
| Non-Hispanic white | 56.30 | 57.59 | 58.06 | 52.77 |  |
| Non-Hispanic black | 13.47 | 10.53 | 13.26 | 17.18 |  |
| Mexican American | 14.33 | 16.79 | 13.34 | 12.56 |  |
| Other race/ethnicity | 15.90 | 15.09 | 15.33 | 17.49 |  |
| PIR | 2.52 ± 1.65 | 2.73 ± 1.64 | 2.57 ± 1.63 | 2.23 ± 1.63 | <0.0001 |
| BMI(kg/m2) | 23.97 ± 6.02 | 24.53 ± 6.38 | 23.90 ± 5.85 | 23.40 ± 5.70 | <0.0001 |
| AST(U/L) | 23.46 ± 10.55 | 22.64 ± 12.00 | 23.68 ± 10.84 | 24.18 ± 8.00 | <0.0001 |
| ALT(U/L) | 19.04 ± 14.22 | 18.73 ± 17.58 | 19.02 ± 11.74 | 19.43 ± 12.23 | 0.3528 |
| Total cholesterol(mg/dL) | 157.24 ± 29.43 | 157.08 ± 28.77 | 157.57 ± 30.26 | 157.05 ± 29.23 | 0.8436 |
| Blood urea nitrogen(mg/dl) | 10.83 ± 3.42 | 10.97 ± 3.49 | 10.66 ± 3.16 | 10.86 ± 3.62 | 0.0244 |
| Serum creatinine(mg/dl) | 0.73 ± 0.19 | 0.70 ± 0.15 | 0.73 ± 0.17 | 0.75 ± 0.25 | <0.0001 |
| Serum cotinine(mg/dl) | 13.96 ± 57.16 | 3.14 ± 21.36 | 12.24 ± 58.14 | 28.68 ± 78.07 | <0.0001 |
| Folate supplements(mcg) | 389.77 ± 284.22 | 391.92 ± 301.16 | 400.12 ± 273.32 | 369.27 ± 269.00 | 0.5290 |
| Serum total folate(ng/ml) | 18.88 ± 8.16 | 19.75 ± 8.24 | 18.86 ± 8.26 | 17.87 ± 7.83 | <0.0001 |

Mean ± SD for continuous variables: the P value was calculated by the weighted linear regression model. (%) for categorical variables: the P value was calculated by the weighted chi-square test. Abbreviation: PIR: Ratio of family income to poverty;BMI: body mass index; AST: Aspartate Aminotransferase;ALT:Alanine Aminotransferase.
